# Modified technique of transforaminal lumbar interbody fusion for segmental correction of lumbar kyphosis: a safe alternative to osteotomies?

**DOI:** 10.1186/s13037-017-0135-z

**Published:** 2017-07-05

**Authors:** Sebastian Weckbach, Heiko Reichel, Michael Kraus, Tugrul Kocak, Friederike Lattig

**Affiliations:** 1 Department of Orthopedics, University of Ulm, Oberer Eselsberg 45, 89081 Ulm, Germany; 2 Spine Center, Berit Paracelsus Clinic, Voegelinsegg 5, 9042 Speicher, Switzerland

**Keywords:** Sagittal imbalance, Osteotomy, Lumbar spine, Transforaminal lumbar interbody fusion

## Abstract

**Background:**

Sagittal rebalancing of a fixated lumbar hypolordosis (kyphosis) is very important to gain satisfactory results. To correct a misalignment vertebral column resection or pedicle subtraction osteotomies are favored, disregarding the relatively high complication rates. The aim of this study was to evaluate the efficiency and safety of a new modified transforaminal lumbar fusion technique as an alternative.

**Methods:**

We conducted a retrospective review (06/2011-06/2015 ) of a prospective database at an University hospital. Inclusion criteria were adult patients with a fixated lumbar hypolordosis and the need of monosegmental correction of more than 10° with an mTLIF. Exclusion criteria consisted of minor aged patients and polysegmental corrections. Study parameters were the perioperative complications and the achieved postsurgical lordosis. The follow up period was 6 months.

**Results:**

A total of 11 patients could be included. The mean segmental lordosis was -2.3° ± 12.4° (range -22° to 14°) preoperative and 15.5° ± 10.5° (range 0° to 29°) postoperative. The degree of correction was 17° ± 5.7° in mean per treated segment (range 12° to 29°). No neurologic or vascular complications occurred. No substantial loss of correction or implant failure was noted during the 6-month follow-up.

**Conclusion:**

The modified transforaminal lumbar fusion technique is a safe method to correct a fixated lumbar kyphosis. The potential of segmental correction is comparable to pedicle subtraction osteotomies but sparing potentially healthy segments.

## Background

Sagittal balance of the spine has been defined as an important factor for patients’ satisfaction and prevention of adjacent segment disease in lumbar fusion surgery [1]. Therefore, reconstruction of a physiological lumbar lordosis is a keypoint in spondylodesis independent of the underlying pathology and the surgical procedure used [2, 3]. Loss of segmental lordosis is mainly due to disc degeneration, discitis or vertebral fracture. The majority of alterated segments remain mobile, which allows the restoration of a lordosis without performing an extended release. Sometimes however the segmental kyphotic deformity is fixed due to large spondylophytes, ossification of the anulus and anterior longitudinal ligament. To restore a physiological lordosis, a circumferential release of the affected segment is necessary which is performed via an anterior-posterior approach. Alternatively a wedge osteotomy like pedicle subtraction osteotomy (PSO) or vertebral column resection (VCR) could be performed [4]. Using these techniques a correction of the sagittal profile and a restoration of lumbar lordosis up to 35 degrees can bei achieved. However going along with a high complication rate especially neural injuries and high intraoperative blood loss [5]. In addition, further potentially healthy segments have to be included in those fusion procedures for stability reasons. In general, anterior-posterior approaches and 3-column osteotomies are associated with high perioperative complication rates of more than 30% [6].

Using the widely established technique of the unilateral transforaminal lumbar interbody fusion (TLIF) for the circumferential release with anterior cage support could help to avoid these typical complications and spare unaffected segments.The purpose of this study was to evaluate the reconstructive potential of lumbar lordosis in fixed kyphotic segments via a modified unilateral transforaminal approach mTLIF.

## Methods

During a four year study period (06/2011 – 06/2015) at a German University hospital consecutively all adult patients fulfilling the inclusion criteria were included in the study. A retrospective review of a prospective database was carried out. Inclusion criteria were adult patients with a fixated monosegmental lumbar hypolordosis undergoing surgical correction of the sagittal profile within the index segment aiming for a correction of more than 10° Cobb angle. Radiological documentation with standard upright X-rays of the lumbar spine was taken preoperatively as well as 1 and 6 weeks and 6 months postoperatively. Children and patients with multilevel pathology were excluded.

Study parameters were the angles of lordosis of the treated segment measured by the Cobb's method on a lateral standing x-ray. Furthermore, periinterventional complications and blood loss were evaluated.

Surgical technique: After exposure of the affected segment from posterior and insertion of the pedicle screws a typical transforaminal approach is performed from one side. The ipsilateral facet joint is resected, whereas on the opposite the joint is only reduced with removing the capsule, remaining cartilage and osteophytes. If necessary the spinal canal could be decompressed in cross-over technique. The remaining disc material is removed as much as possible. Small curved curretts and rongeurs are helpful for this step. If the disc space is very small a chisel could be used as well. The release of the lateral and anterior structures starts from the already performed incision of the ipsilateral annulus. The descending nerve root is protected and the lateral annulus is dissected in anterograde direction with a sharp instrument, e.g. an 8 mm chisel. As soon as some mobility of the segment is achieved, a posterior distraction of the segment via the pedicle screws or the posterior spinous process is performed. Then the anterior part of the annulus with the anterior longitudinal ligament is dissected either with a punch or a chisel cutting backwards starting as far as possible on the contralateral side. Afterwards a plain spreader is inserted between the vertebral bodies. With a dosed force the segment is spread until the remaining posterolateral fibres of the contralateral annulus are torn and the segment is completely mobile.

The study protocol fulfilled the requirements by the University Ethics Committee Ulm, Germany and was approved. The statistics were descriptive and results presented in mean values.

## Results

Regarding the inclusion and exclusion criteria overall 11 patients were eligible for a monosegmental correction using the mTLIF. Out of these patients, seven were female and four male with a mean age of 57 years (range 42 to 78 y). Five patients had previous lumbar spine surgery. Indication for surgery was mainly degenerative disc disease. The level of the surgical intervention was in one case L1/2, in two cases L2/3 and in four cases L3/4 and L4/5. The patient specific characteristics were shown in Table 1.

**Table 1:** Patients demographics: Patients age, treated segments, diagnosis and past surgical history

Pat	Age	Gender	Level mTLIF	Preop. diagnosis	Previous surgery
1	67	m	L4/5	Osteochondrosis	none
2	46	f	L4/5	Osteochondrosis	Decompression same level
3	78	f	L3/4	Osteochondrosis	Laminectomy L4
4	56	f	L3/4	Deg. kyphoscoliosis	none
5	45	f	L4/5	Osteochondrosis	none
6	69	m	L2/3	Iatrogenic kyphosis with fracture	Verteboplasty, Laminectomy
7	48	m	L3/4	Lytic spondylolisthesis	none
8	42	f	L3/4	Osteochondrosis	Distraction fusion L4-S1
9	73	m	L4/5	Deg. kyphoscoliosis	none
10	65	m	L2/3	Osteochondrosis	Fusion L3-S1, Neurostimulator
11	42	f	L1/2	Fracture L1	none

**Fig. 1:**
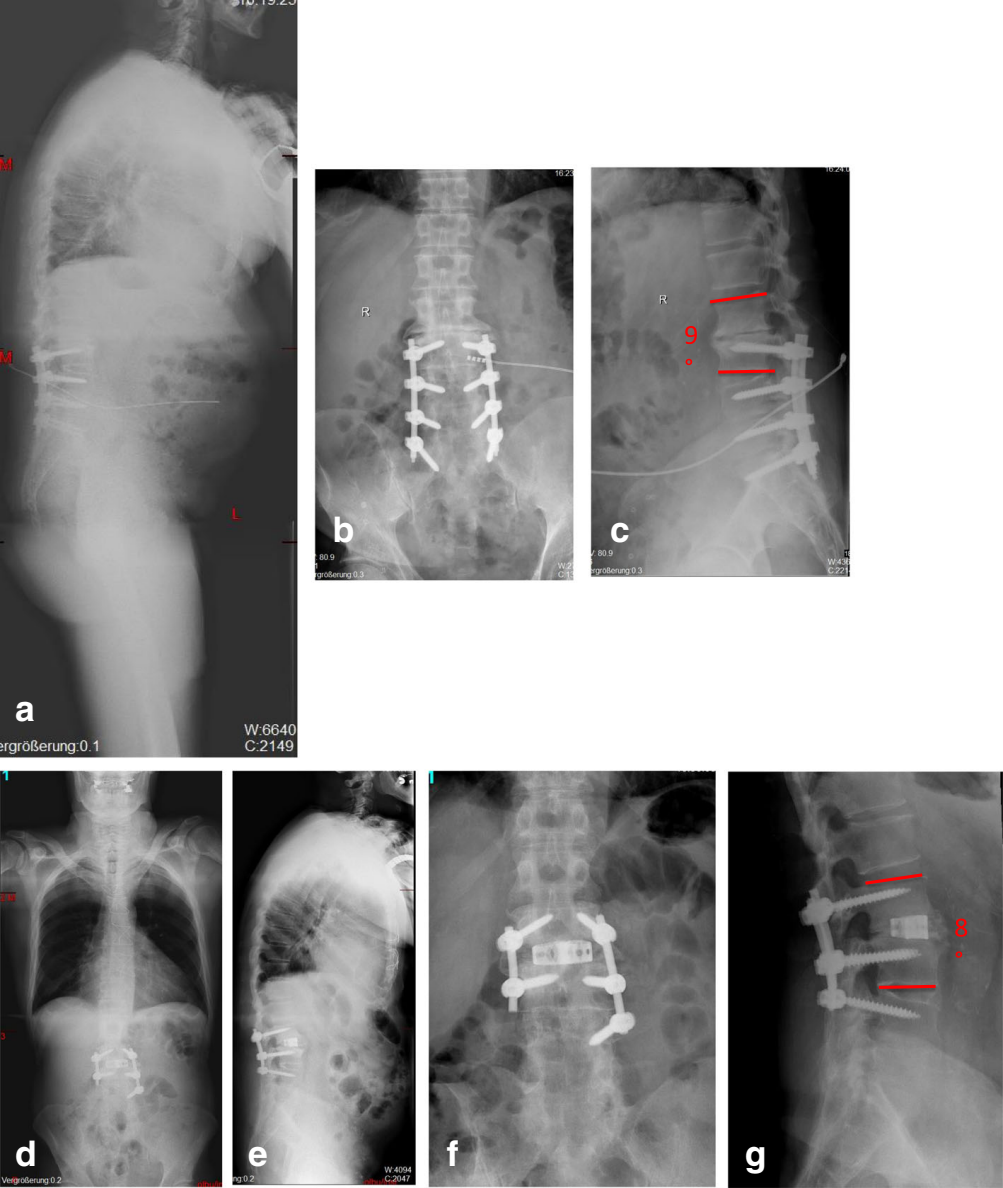
65- year old male with fixed adjacent segment disease L2/3 post posterior instrumentation and fusion L3-S1 including the implantation of an neurological stimulator in an outside hopsital. Figure **a** shows the sagittal whole-spine x-ray, Figure **b** the standing ap x-ray of the lumbar spine, Figure **c** the lateral x-ray including the measurements of the deformity. Figures **d** and **e** display the postop standing whole-spine x-rays, Figures **f** and **g** the lumbar x-rays including the measurements

**Fig. 2:**
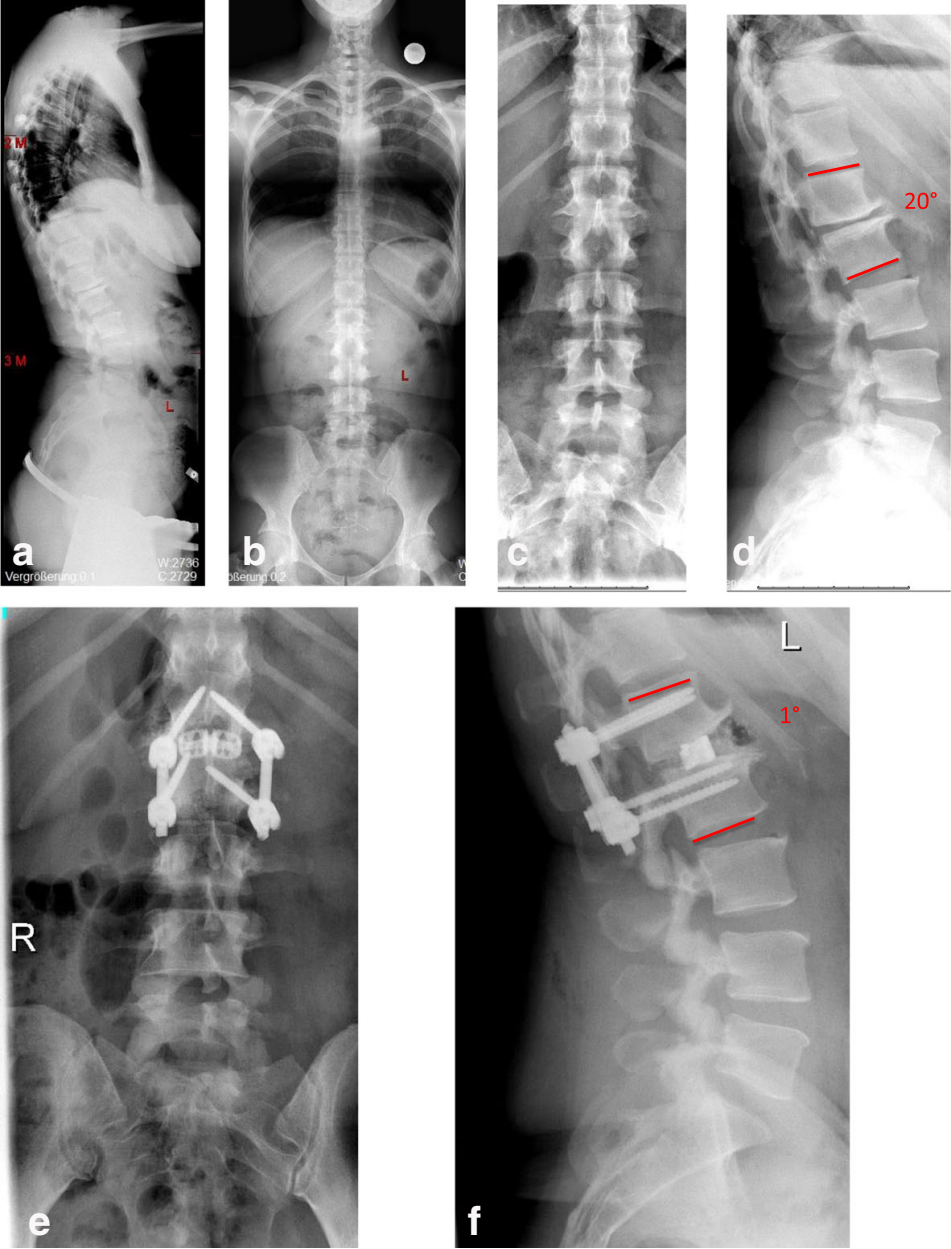
42- year old female with status post L1 fracture and segmental kyphosis L1/2. Figures **a**/**b** display the whole spine x-rays, Figures **c**/**d** the lumbar x-rays in both planes including the measurements of the deformity. Figures **e**/**f** show the postop standing x-rays of the lumbar spine including the measurements in both planes

The mean segmental lordosis was -2.3° ± 12.4° (Range -22° to 13° Cobb-angle) preoperatively. One week after mTLIF the mean segmental lordosis was measured with 15.5° ± 10.5° (Range 0° - 28°). These results could be confirmed at the 6- month follow- up with a mean segmental lordosis of 14.7° ± 10.7 (0° to 28°). The correction potential of the mTLIF with the 270° release was in mean 17° ± 5.7° (Range 12° to 29°). Loss of segmental lordosis between one week and 6 months postoperatively was 0.7° (Range 0° to 3°). The results are shown in Table 2 in detail. Exemplary for the surgical intervention, clinical cases are shown in Figs. 1 and 2.

**Table 2:** Surgical implants: Cage type, absolute and mean measures ± SD and correction for lumbar lordosis (LL) and lordosis of the mTLIF segment (SL)

Pat	Cage	SL preop	SL 1 week postop	SL 6 mo postop	Correction mTLIF segment
1	Banana 6°	-6°	6°	6°	12°
2	Mesh 0°	14°	26°	26°	12°
3	Banana 10°	3°	17°	16°	13°
4	Mesh 0°	0°	13°	12°	12°
5	Banana 10°	12°	26°	26°	14°
6	Mesh 0°	-18°	0°	0°	18°
7	Banana 6°	-5°	12°	11°	16°
8	Mesh 28°	-2°	27°	27°	29°
9	Banana 10°	13°	29°	28°	15°
10	Banana 10°	-14°	13°	10°	24°
11	Banana 6°	-22°	1°	0°	22°
		-2.3° ± 12.4°	15.5° ± 10.5°	14.7° ± 10.7°	17° ± 5.7°

Regarding complications, neither major complications such as major vessel injury or neurologic deficit nor minor complications like wound infections occured. The 6-month follow- up didn’t reveal any signs of implant failure or screw loosening.

## Discussion

The importance of sagittal balancing of the spine is getting more and more into focus. Restoration of the sagittal balance reduces pain and improves gait efficiency [7]. Fixed sagittal deformities are corrected generally using different osteotomies like PSO or VCR. We hypothetized that fixed spinal sagittal deformities could also be adressed using the above described modification of the commonly known TLIF procedure. Using this technique, almost 270° release of the disc anulus can be achieved even in fixed segments. Therefore, the mTLIF is a safe alternative procedure for rebalancing the lumbar spine in selected indications. Our data revealed a correction potential of up to 25° per segment.

Correction of deformities using osteotomies requires additional fixation of potentially healthy segments. For PSO Berjano et al. [4] recommended to extend the fixation two levels above and two levels below the index segment. For PSO, a correction of 17.9° +/- 4.3° is described [8]. Furthermore, the bilateral approach and the closing wedge resection in PSO may be as well causative for the high risk of neurologic complications. Complications in PSO are described in literature ranging from 4 to 15% [9–11].

In patients with remaining mobility in unaffected lumbar segments, a fixation of these segments should be avoided whenever possible. Any type of reconstructing lumbar lordosis by spinal osteotomy involving the anterior column cannot be fused monosegmentally. Using the mTLIF technique a single level spondylodesis including the segmental restoration of lordosis can be achieved. In our series the patients were treated solely monosegmentally without implant failure. In one case, a slightly loss of correction of 3° after 6 months was observed. Nevertheless, a higher loss of correction and subsidence of the cages during the follow-up period of 6 months was not documented. This technique could even be simplified using an expandable model of TLIF - cage with a variety of lordotic angles up to 25°. If the difference between the segmental lordosis and the cage lordosis increases, the contact area of the cage and the vertebral endplates is diminished with increasing risk for subsidence of the cage. Different studies support that cages with larger bearing surface show less subsidence [12–14].

The main working zone of the mTLIF technique is the disc space. Larger spondylophytes and a partially ossificated disc could be addressed with the technique as shown. To benefit from the advantages, particularly the estimated minor blood loss and the possibly lower risk for neurologic complications, the authors do not recommend the technique in segments with totally ossification of the disc e.g. as in patients with ankylosing spondylitis. Furthermore, in cases requiring a segmental lordosis of more than 25°, the mTLIF technique is at its limit and the classical osteotomies such as PSO should be used. Whether a polysegmental mTLIF could be an alternative to vertebral osteotomies in those cases is currently under evaluation.

This study is, to the best of the authors knowledge, the first description of the surgical technique and evaluation of patients treated with single level mTLIF. Certainly, the study cohort was small, the investigation was not designed as a matched control study and the patients were not randomized. The follow- up period of 6 months was short but in context with the literature, the occurrence of radiolucent zones around pedicle screws as a sign of a developing pseudarthrosis is at its peak about 6 months postoperatively [15, 16]. Hence, the period seems to be sufficient to evaluate, if the mTLIF construct provides enough stability and is comparable to instrumentations in bony osteotomies.

## Conclusion

The mTLIF technique is a safe and effective method correcting segmental lumbar hypolordosis up to 25°. The less extensive approach compared to bony osteotomies can help to reduce blood loss and spare potentially healthy segments. Therefore, the mTLIF should be considered as an alternative to a PSO in selected cases.
